# Effect of emotional intelligence on problematic mobile social media use: mediating role of peer relationships and experiential avoidance

**DOI:** 10.3389/fpsyg.2025.1558733

**Published:** 2025-06-19

**Authors:** Xin Chen, Sifan Peng, Hexu Guan, Huanran Sun, Hongxuan Wu, Xumei Yao, Zi Chen, Xi Yang

**Affiliations:** School of Psychology, Chengdu Medical College, Chengdu, China

**Keywords:** emotional intelligence, peer relationship, experiential avoidance, problematic mobile social media use, internet addiction

## Abstract

**Objective:**

The I-PACE model was employed as the theoretical framework to investigate the effect of emotional intelligence on problematic mobile social media use. Furthermore, this study explored whether peer relationships and experiential avoidance serve as mediating factors in this relationship.

**Methods:**

A total of 1,448 students from nine public universities across Chengdu, Beijing, Shanghai, and Kunming were recruited between May 1, 2021, and October 28, 2021, through convenience sampling. The survey instruments included the Emotional Intelligence Scale, the Peer Relationship Scale, the Acceptance and Action Questionnaire Second Edition, and the Problematic Mobile Social Media Use Assessment Questionnaire.

**Results:**

(1) Emotional intelligence exhibited a negative correlation with both experiential avoidance and problematic mobile social media use, while it demonstrated a positive correlation with peer relationships; (2) Peer relationships were negatively correlated with both experiential avoidance and problematic mobile social media use, while experiential avoidance was positively correlated with problematic mobile social media use; (3) Peer relationships and experiential avoidance acted as mediators in this relationship, forming a sequential mediation chain and collectively influencing how emotional intelligence affects problematic mobile social media use.

**Conclusion:**

This study investigates the relationship between emotional intelligence and the severity of problematic mobile social media use among Asian participants. Peer relationships and experiential avoidance independently mediate and sequentially mediate the relationship between emotional intelligence and problematic mobile social media use. Emotional intelligence positively influences peer relationships, which subsequently negatively affects experiential avoidance, ultimately reducing problematic mobile social media use. Both peer relationships and experiential avoidance are shaped by emotional intelligence and further contribute to an individual’s problematic mobile social media use.

## 1 Introduction

Currently, mobile online social networks are considered an important medium for communication and connection with others (Chang et al., 2022). The emergence of these online platforms that integrate functions such as personal status updates, browsing friends’ statuses, consuming news, and forming interest groups (similar to applications like Facebook, TikTok, QQ, WeChat, and Microblog) has fundamentally changed the way humans communicate (Lim, 2023). Due to the characteristics of immediacy, rapidity, and the ability to transcend spatial limitations inherent in mobile online social networks, these platforms can more effectively address specific psychological needs of individuals, such as the need for intimacy and social interaction (Jo and Baek, 2023).

As technology continues to advance, individuals are increasingly engaging with online social networking platforms through mobile devices such as smartphones and tablets, which provide enhanced mobility and convenience compared to conventional PC terminals. The utilization of online social networks through mobile smart devices has significantly gained popularity in the daily lives of individuals in contemporary society (Arrivillaga et al., 2022).

Based on the 54th Statistical Report on China’s Internet Development, by August 2024, The user base of the Internet in China had expanded to 1.092 billion. Of these, nearly all individuals (99.9%) use mobile devices to access the internet. Within the college student demographic, almost every individual possesses at least one social network account (China Internet Network Information Center, 2024).

Compared to PC terminals, mobile smart devices have significantly reduced barriers for individuals to engage in online social networks. The portability and ease of use of these devices enable users to connect, exchange information, and learn anytime and anywhere.

However, while smart mobile devices offer enhanced convenience and benefits in individuals’ lives, they also lead to excessive engagement in online social networks, such as prolonged late-night browsing of social websites, uncontrolled consumption of short videos, incessant chatting, and endless scrolling through microblogs, may potentially lead to problematic usage or addiction (Satici, 2019). When an individual develops problematic mobile social media use (PSMU) due to excessive use of mobile social media, even though it may not yet meet the clinical diagnostic criteria for addictive behavior, the aforementioned manifestations and conditions have already emerged and negatively affected their daily life, studies, and work.

PSMU is a subtype of internet addiction that has evolved from traditional internet addiction. It describes a psychological and social phenomenon wherein individuals, following prolonged exposure to highly engaging social media, find it challenging to moderate their screen time. This excessive consumption of information frequently escalates into a state of mental and physical discomfort, resulting in feelings of being overwhelmed and disoriented (Andreassen and Pallesen, 2014). The distinction between PSMU and regular or frequent utilization of mobile social media lies in its inherent lack of regulation, compulsiveness, and propensity for negative outcomes. These adverse consequences are closely associated with the usage of such platforms (Swanton et al., 2021).

## 2 Emotional intelligence and PSMU

Social mobile media serves as a sociocultural instrument to address various psychological needs (Andreassen, 2015). However, if individuals rely solely on mobile social media platforms to fulfill their core psychological needs, they may exhibit a range of concerning usage patterns in online social networking. This over-reliance can result in behaviors that are potentially detrimental or unhealthy in online social interactions (Andreassen, 2015). Individuals who excessively engage in mobile social media may exhibit symptoms similar to addiction, such as impaired emotional regulation, weakened executive control, increased susceptibility to attentional bias, and reduced academic performance (Weinstein, 2023; Stockdale and Coyne, 2020; Gugushvili et al., 2024; Qi et al., 2024; Ulvi et al., 2022; Shiraly et al., 2024; Hashemi et al., 2022). Given the high prevalence and adverse consequences of PSMU, researchers are increasingly focusing on identifying risk factors and protective mechanisms associated with PSMU, such as emotional intelligence. As a personality trait closely associated with emotional characteristics, emotional intelligence (EI) may play a significant role in influencing the formation and development of an individual’s PSMU.

EI is typically described as the capacity to accurately identify, manage, and express individual emotions, and to perceptively understand and respond to the emotions of others. As a personality trait primarily characterized by emotional features (Andrei et al., 2016), this trait enables individuals to navigate their surroundings with greater ease, fostering both personal growth and a sense of balance in their lives. This study aims to explore the relationship between EI as an individual trait and PSMU.

Based on the Interaction of Person-Affect-Cognition-Execution (I-PACE) theory (Brand et al., 2016), whether it is traditional internet addiction or more particular forms of problematic online behavior such as PSMU, the development and manifestation of their symptoms are outcomes resulting from the interplay between individual personality traits, affective states, cognitive processes, and behavioral patterns. As a fundamental component of personality traits and a crucial determinant of emotions, EI may serve as a significant determinant for individuals’ propensity to develop PSMU and other internet-related issues (Brand et al., 2019). Research has demonstrated a consistent association between lower levels of emotional intelligence and the presence of substance addiction (Zhang, 2023), as well as issues related to addiction, including problem gambling (Richard and King, 2023), excessive internet use (Alshakhsi et al., 2022), smartphone overuse (Aranda López et al., 2022), and compulsive online gaming (Wang et al., 2022). Individuals may adopt addictive behaviors as maladaptive coping mechanisms that ultimately cause more harm than good when dealing with negative emotions (Wang J. et al., 2024; Wang Y. et al., 2024). Individuals who experience difficulties with emotion regulation may be more likely to perceive PSMU as a coping strategy (Flack et al., 2024).

Therefore, it is crucial to reveal how EI influences PSMU in order to elucidate the characteristics and mechanisms associated with individual problematic internet use. To summarize, this research proposes H1: EI has a significant negative effect on PSMU.

However, the intricate interplay among emotions, personality traits, and cognitive factors collectively influences the relationship between EI and PSMU. Previous research has demonstrated that peer relationships have a significant influence as an affective factor on internet addiction among adolescents (Erdem and Sezer Efe, 2022), while experiential avoidance plays a vital role as a cognitive factor influencing internet addiction (Kim and Bae, 2022).

## 3 Peer relationships and experiential avoidance

Peer relationships (PR) describe the emotional and psychological bonds formed between individuals within the same age range or peer groups, often as a result of shared interests, hobbies, or similar attributes. These interpersonal relationships are formed and strengthened through interactions among peers who share similar ages or levels of psychological maturity (Shao et al., 2024). In contrast to vertical interpersonal relationships, such as the familial bonds between parents and children or the interactions between teachers and students, PR represent a distinct form of parallel interpersonal connections, which are individuals’ earliest experiences with this type of relationship (Geukens et al., 2022). Prior study has shown a significant association between PR and EI (Ferguson and Ryan, 2019). PR can provide adolescents with abundant social and emotional support, fulfill their interpersonal relationship needs, alleviate the negative effects of stressful events on them, thus promoting positive emotional experiences and behavioral responses in individuals (Bae et al., 2022). The satisfaction of individual PR and interpersonal interaction needs should be grounded in authentic social relationships and emotional interactions. However, with the gradual emergence of online social networking services as a significant means to establish, develop, and maintain interpersonal connections, coupled with the proliferation of mobile and convenient smart devices, individuals tend to increasingly confine their PR and interpersonal interactions within the virtual realm of social networks. In other words, individuals’ PR and interpersonal connections are progressively established through online social networking services. This phenomenon may further contribute to the occurrence of PSMU in individuals (Salehi et al., 2021). EI has a direct effect on the quality of PR (Barragán Martín et al., 2021). The perceived PR of individuals, as an emotional factor, may constitute a crucial element in explaining the association between individual personality traits, such as EI, and coping styles, such as PSMU. Earlier studies have also indicated that reduced EI tends to coincide with diminished quality of PR (Ferguson and Ryan, 2019). Moreover, the previous studies reveal that PR was negatively correlated with PSMU (Chen et al., 2023; Koc and Gulyagci, 2013). Therefore, drawing upon the evidence presented, it can be inferred that PR have a significant influence on the association between EI and PSMU. In conclusion, this research proposes H2: PR play a mediating role in the relationship between EI and PSMU. However, individuals with relatively low levels of EI and poor PR often lack adequate social and emotional support, which hinders their ability to effectively manage the negative emotions and adverse events they encounter. Simultaneously, these individuals exhibit a strong desire for social belonging and a tendency to escape from reality as a means of avoiding unpleasant experiences and events. This propensity drives them to seek refuge in the virtual online social media world, where they utilize PSMU as a coping mechanism to evade real-life pressures, confront negative events and emotions, and attain alternative forms of satisfaction. In this transformation process, experiential avoidance (EA) may serve as a mediating factor that influences the relationship between PR and PSMU.

EA describes an individual’s resistance to experiencing or attempting to eliminate specific internal experiences, including emotions, thoughts, bodily sensations, memories, and behavioral tendencies. Subsequently, individuals endeavor to employ corresponding strategies in order to modify these experiences and the contextual factors that give rise to them (Hayes et al., 1996). Similarly, EA can function as a key emotional and cognitive factor that helps explain the underlying reasons for PSMU. EA is deeply ingrained in the fundamental cognitive behavioral patterns of human beings, thereby exerting extensive permeability and influence on individual cognitive processes and behavioral responses (Baykan and Can, 2023). A wide range of psychopathological phenomena, including Emotional Apathy, Dissociative Disorders, and Specific Phobia, can be conceptualized as maladaptive strategies stemming from experiential avoidance, addictive behaviors also fall into this category (Na et al., 2022). Based on the concept of EA, Cavicchioli et al. (2022) proposed a novel perspective on addiction known as the self-centered EA model. This model proposes that addiction functions both as a coping strategy for maladaptive individuals to avoid certain experiences and as an interactive mechanism that facilitates their engagement with the social environment. EA enables flexible regulation of an individual’s addictive behavior in terms of intensity and extent. However, as a typical cognitive response pattern and coping mechanism, EA often only has short-term effects and does not yield positive long-term outcomes. In fact, the results may even be detrimental (Wang et al., 2024). Earlier studies have indicated that individuals with higher EI tend to exhibit lower tendencies toward engaging in EA (Zhou et al., 2022). In essence, heightened EI corresponds to reduced resistance or inclination to eliminate specific emotions within oneself. EA, as an emotional cognitive factor and coping mechanism, can partially elucidate the propensity of maladaptive individuals to excessively rely on online social networking services when confronted with challenging situations. EA has been identified in previous studies as one of the predictive factors for PSMU (Faghani et al., 2020). In conclusion, H3 is proposed: EA serves as a mediator in the relationship between EI and PSMU.

The intervention study conducted by Wolgast et al. (2013) indicates that implementing positive interventions targeting individual PR can significantly influence levels of EA, which in turn indirectly regulates individuals’ negative emotions. Hence, PR play a crucial role in predicting an individual’s propensity for EA. Individuals with lower-quality PR also tend to exhibit relatively deficient self-emotional regulation abilities. When confronted with unpleasant stimuli or situations, they frequently resort to avoidance or escape as a coping mechanism (Herd and Kim-Spoon, 2021). Virtual online social networks offer them a comparatively secure and cost-effective environment, thereby making immersion in online social networks their preferred means of evading or eluding distressing stimuli or situations (García Montes et al., 2024). Hence, it can be inferred that EA potentially mediates the association between PR and PSMU. In light of this, H4 is posited in the present study: The relationship between EI and PSMU will be sequentially mediated by PR and EA.

## 4 The present study

**Figure 1:**
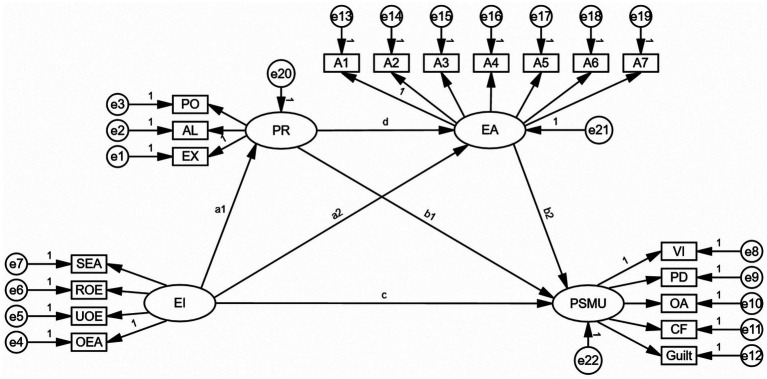
Theoretical model. SEA, Self-emotional assessment; UOE, use of emotion; OEA, assessment of others’ emotions; and ROE, regulation of emotion; PO, popularity; AL, aloneness; EX, exclusion; EA, Experiential avoidance, the dimensions of experiential avoidance encompass A1-A7. VI, viscosity increase; PD, physiological damage; OA, omission anxiety; CF, cognitive failure.

Existing literature has discussed the relationship between EI and PSMU. However, the mediating mechanisms that link EI and PSMU have frequently been neglected in the existing literature. Exploring the relationships and mechanisms among EI, PR, EA, and PSMU can expand theoretical horizons and offer novel insights for the advancement of addiction model theories and the clinical intervention of internet addiction disorder. At the foundational theoretical level, such exploration facilitates the establishment of a development-formation-maintenance loop model for social media addiction, thereby enriching and refining the research domain of problematic internet use. At the clinical application level, it provides educational, rehabilitative, and medical institutions with valuable guidance and actionable strategies to address individuals’ problematic internet use and addiction, particularly concerning online social media. This not only mitigates the adverse consequences of PSMU but also introduces innovative approaches, perspectives, and methodologies for preventing and managing psychological distress, issues, and disorders associated with internet use. Therefore, this study, grounded in the I-PACE model, aims to explore the mechanisms by which PR and EA serve as mediators in the relationship between EI and PSMU. As illustrated in Figure 1, the theoretical model is presented.

## 5 Methods

### 5.1 Participants

In this research, data were collected via the offline distribution of paper-based questionnaires from May 1, 2021 to October 28, 2021. This research received approval for human research protection from the Ethics Review Committee of Chengdu Medical College (approval number: 2021NO.07), in strict adherence to the guidelines outlined in the Declaration of Helsinki. Prior to completing the questionnaire, participants were provided with detailed information regarding the study. Additionally, they were assured that their responses would be treated with strict confidentiality and anonymity. The informed consent statement was incorporated into the distributed paper questionnaire in written form. In the study, the completion and submission of the questionnaire by the participants were regarded as indicating informed consent and voluntary participation. The content of the informed consent statement in the questionnaire is as follows (S1 File): “If you confirm that you have understood the content and purpose of this research survey and agree to participate, please complete this questionnaire based on your true situation. If you refuse to participate, please do not complete the questionnaire and return it.”

Participants for this study were recruited through a convenience sampling method. 1,448 questionnaires were administered across nine public universities located in four Chinese cities: Chengdu, Beijing, Shanghai, and Kunming. The standardized tests were conducted with the on-site guidance of the test facilitator, resulting in a collection of 1,419 valid questionnaires (50.8% male, 49.2% female), thereby achieving an effective recovery rate of 98.0%. The mean ages were 20.94 ± 2.06 years for the overall sample, 21.39 ± 2.11 years for male participants, and 20.48 ± 1.90 years for female participants.

### 5.2 Measures

After evaluating the aforementioned demographic characteristics, the following psychological scales were subsequently administered.

### 5.3 Problematic mobile social media use assessment questionnaire (PSMU-Q)

The assessment of PSMU was conducted through the PSMU-Q (Jiang, 2018). This tool consists of 20 concise questions designed to evaluate five critical dimensions of PSMU: cognitive failure (CF), omission anxiety (OA), viscosity increase (VI), physiological damage (PD) and guilt. Responses are rated using a 5-point Likert scale, with options ranging from 1 (totally disagree) to 5 (totally agree). Higher scores indicated more severe PSMU. The Cronbach’s alpha coefficient for this scale was calculated to be 0.878. In this study, the structural validity of the questionnaire was evaluated through confirmatory factor analysis (CFA), and the results indicated an acceptable model fit: *χ*^2^/*df* = 4.347, GFI = 0.955, TLI = 0.952, SRMR = 0.051, RMSEA = 0.049.

### 5.4 Emotional intelligence scale-C (WLEIS-C)

EI was evaluated using the WLEIS-C (Wong and Law, 2002; Di et al., 2022). This instrument comprises 16 brief items that assess four dimensions of EI: Self-emotional assessment (SEA), assessment of others’ emotions (OEA), use of emotion (UOE) and regulation of emotion (ROE). Responses are rated using a 7-point Likert scale, with options ranging from 1 (totally disagree) to 7 (totally agree). Higher scores reflecting greater EI. The Cronbach’s alpha coefficient for this scale was calculated to be 0.865. In this study, the model fit of the scale was acceptable: *χ*^2^/*df* = 5.538, GFI = 0.954, TLI = 0.952, SRMR = 0.054, RMSEA = 0.057.

### 5.5 Acceptance and action questionnaire second edition (AAQ-II)

EA was evaluated using the validated Chinese version of the AAQ-II (Hu et al., 2021; Bond et al., 2011). This instrument comprises 7 brief items that assess EA with options ranging from 1 (totally disagree) to 7 (totally agree). Higher scores reflecting more severe experiential avoidance. In this sample, The Cronbach’s alpha coefficient for this scale was calculated to be 0.893. In this study, the model fit of the scale was acceptable: *χ*^2^/*df* = 1.603, GFI = 0.995, TLI = 0.997, SRMR = 0.011, RMSEA = 0.021.

### 5.6 Peer relationship scale-C (PRS-C)

PR were evaluated using PRS-C (Zhang et al., 2021; Asher et al., 1984). This tool consists of 16 concise questions designed to evaluate three critical dimensions of PR: popularity (PO), aloneness (AL), exclusion (EX). Responses are rated using a 4-point Likert scale, with options ranging from 1 (totally disagree) to 4 (totally agree). Higher scores indicate enhanced peer relationships. The Cronbach’s alpha coefficient for this scale was calculated to be 0.908. In this study, the model fit of the scale was acceptable: *χ*^2^/*df* = 7.953, GFI = 0.944, TLI = 0.938, SRMR = 0.059, RMSEA = 0.070.

### 5.7 Procedures

For this research, data collection was conducted through the distribution and immediate on-site collection of completed paper questionnaires, thereby ensuring the quality and integrity of the questionnaire data. Two postgraduate students specializing in psychology, along with a psychological officer, collaboratively conducted the questionnaire survey. Before distribution, the two psychology graduate students gave standardized verbal instructions, explained the questions raised by the participants, and ensured uniform collection procedures thereafter. The survey included an informed consent section, participant instructions, specific requirements, and key precautions. Questionnaires containing incomplete entries or unanswered questions were considered invalid. From the 1448 collected questionnaires, 1,419 valid responses were ultimately obtained (98.0% response rate).

### 5.8 Data analysis

Data analysis was conducted using SPSS 23.0. To evaluate gender differences in the variables, independent samples t-tests were conducted. Subsequently, a Pearson correlation analysis was performed. Furthermore, Harman’s single-factor test was utilized to evaluate the common-method bias. Since all measurements were obtained from self-report questionnaires, if exploratory factor analysis reveals that a single factor accounts for the majority of the variance, this may indicate the presence of common-method bias (Podsakoff et al., 2003). Structural equation modeling (SEM) was conducted using AMOS 23.0. In this research, the accuracy and reliability of each measurement instrument were rigorously assessed. When Cronbach’s *α* is greater than 0.60, it is considered acceptable; when it exceeds 0.70, it is regarded as good (Churchill, 1979). To assess the structural validity, CFA was utilized. The reported model fit statistics included: TLI > 0.80, GFI > 0.80, SRMR < 0.08, RMSEA < 0.08 and 1 < *χ*^2^/*df* < 8 (Fornell and Larcker, 1981; Doll et al., 1994; Hooper et al., 2008). If all aforementioned conditions are fulfilled, the structural validity can be deemed satisfactory. The mediation and confidence intervals were estimated using the non-parametric percentile Bootstrap method with bias correction. Bias-corrected 95% confidence intervals (CI) were calculated utilizing 5000 bootstrap samples. Statistical significance was established if the 95% CI did not include zero. In this study, statistical significance was determined using a threshold of *p* < 0.05, and the scoring criteria were established as follows: 1 < *χ*^2^/*df* < 3 (good model); 3 < *χ^2^*/*df* < 5 (acceptable); AGFI, CFI, NFI, and GFI > 0.90; RMSEA ≤ 0.05, indicating a good fit; 0.05 < RMSEA < 0.08, suggesting an acceptable fit (Hau et al., 2004).

## 6 Results

### 6.1 Common-method bias

Harman’s single-factor test was utilized to evaluate the common-method bias (Podsakoff et al., 2003). The exploratory factor analysis revealed that 13 factors had eigenvalues higher than 1, with the primary factor accounting for 20.58% of the total variance. Common-method bias is not present in this study.

### 6.2 Preliminary analyses and gender differences

Table 1 presents a summary of the means, standard deviations, and gender-based differences. There were no statistically significant differences in the scores pertaining to EI, PR, EA, and PSMU between participants of different genders (*p* < 0.05).

**Table 1:** Gender differences.

Statistical variable	Variables	EI (M ± SD)	PR (M ± SD)	EA (M ± SD)	PSMU (M ± SD)
Gender	Female (*n* = 698)	67.07 ± 14.48	40.45 ± 9.58	23.86 ± 9.56	56.75 ± 12.75
	Male (*n* = 721)	67.46 ± 15.35	40.74 ± 9.68	25.59 ± 9.50	56.69 ± 12.67
	*t*	0.48	0.56	−1.36	−0.87

*denotes *p* < 0.05. *N* = 1419.

### 6.3 Correlation analysis

The descriptive statistics and Pearson correlation coefficients for the variables, as detailed in Table 2. There were significant correlations among EI, PR, EA, and PSMU. EI was positively correlated with PR, while EA was positively correlated with PSMU. The remaining variables exhibit significant negative correlations with each other.

**Table 2:** Correlations between variables.

Variables	M	SD	1	2	3	4
1. EI	67.27	14.92	1.00	–	–	–
2. PR	40.60	9.63	0.33^***^	1.00	–	–
3. EA	26.00	8.28	−0.23^***^	−0.28^***^	1.00	–
4. PSMU	56.72	12.70	−0.36^***^	−0.38^***^	0.59^***^	1.00

*N* = 1419. *** Denotes *p* < 0.001.

### 6.4 Mediating effect test of the overall model and exploring the mediation model

A SEM was constructed utilizing AMOS 23.0, wherein PSMU served as the dependent variable, EI as the independent variable, and PR and EA acted as mediating variables. After completing the model development, a final model exhibiting a satisfactory fit was obtained. The goodness-of-fit statistics are detailed in Table 3.

**Table 3:** Model fit comparison.

Model	RFI	GFI	AGFI	CFI	NFI	TLI	RMSEA	*χ^2^/df*
Model	0.957	0.979	0.973	0.981	0.964	0.977	0.028	2.087
Indices	> 0.90	> 0.90	> 0.90	> 0.90	> 0.90	> 0.90	< 0.05	1 < *χ^2^/df < 3*
	Good	Good	Good	Good	Good	Good	Good	Good

**Figure 2:**
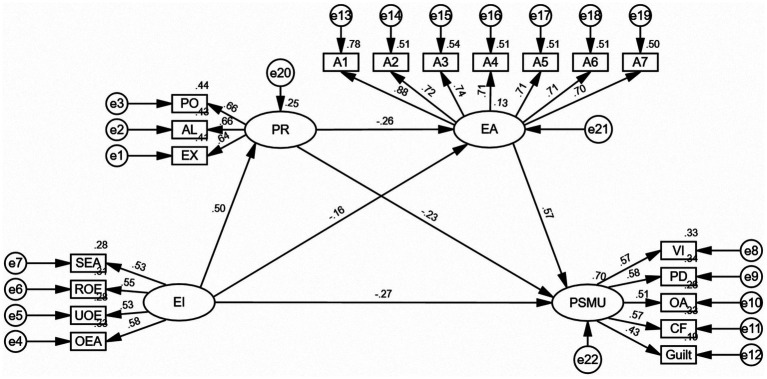
The mediating relationships model of PR and EA between EI and PSMU (Standardization). SEA, Self-emotional assessment; UOE, use of emotion; OEA, assessment of others’ emotions; and ROE, regulation of emotion; PO, popularity; AL, aloneness; EX, exclusion; EA, Experiential avoidance, the dimensions of experiential avoidance encompass A1-A7. VI, viscosity increase; PD, physiological damage; OA, omission anxiety; CF, cognitive failure.

The final model (Figure 2) demonstrated that EI exerts a significant negative effect on PSMU (*β* = −0.27, SE = 0.037, *p* < 0.001), consistent with H1, additionally, it were significantly and positively linked to PR (*β* = 0.50, SE = 0.039, *p* < 0.001), and negatively linked to EA (*β* = −0.16, SE = 0.022, *p* < 0.001). The PR exhibited a significant negative association with EA (*β* = −0.26, SE = 0.027, *p* < 0.001), as well as a significant negative association with PSMU (*β* = −0.23, SE = 0.044, *p* < 0.001). EA exhibited a significant positive association with PSMU (*β* = 0.57, SE = 0.062, *p* < 0.001).

Hence, EI exerts its effect on PSMU through three mediating pathways. The mediating effect was further assessed by means of the bias-corrected percentile bootstrap method, with 5,000 resampled datasets. Pathway 1: EI → PR → PSMU [indirect effect = −0.116, BootSE = 0.021, 95% CI (−0.163, −0.081)]. Pathway 2: EI → EA → PSMU [indirect effect = −0.092, BootSE = 0.024, 95% CI (−0.140, −0.044)]. Pathway 3: EI → PR → EA → PSMU [indirect effect = −0.073, BootSE = 0.013, 95% CI (−0.104, −0.050)]. Furthermore, the 95% bootstrap CI did not include zero, confirming statistical significance. As shown in Table 4, all mediating effects reached statistical significance. First, PR partially mediated the relationship between EI and PSMU, thereby supporting H2. Second, EA also partially mediated the relationship between EI and PSMU, thereby supporting H3. Third, PR and EA sequentially mediated the path from EI to PSMU, thereby supporting H4.

**Table 4:** Bootstrapping effect for the model.

Effect types	Effect	SE	Bootstrapping (95% CI)	
			LLCI	ULCI
Total effect	−0.555	0.035	−0.623	−0.487
Direct effect	−0.274	0.039	−0.352	−0.199
Total indirect effect	−0.280	0.028	−0.341	−0.229
EI→PR→PSMU	−0.116	0.021	−0.163	−0.081
EI→EA→PSMU	−0.092	0.024	−0.140	−0.044
EI→PR→EA→PSMU	−0.073	0.013	−0.104	−0.050

*N* = 1419. SE, Standard Error; LLCI, 95% lower limit CI; ULCI, 95% upper limit CI.

## 7 Discussion

This research aimed to provide deeper insights into the underlying mechanisms associated with the emergence of technology-related issues. In particular, this study utilized a sequential mediation chain model to investigate the predictive factors of PSMU. These findings offer supporting evidence indicating that lower EI was associated with poorer PR, which subsequently contributes to increased EA, ultimately leading to higher levels of PSMU.

According to the I-PACE model, EI, as an integral component of an individual’s personality and emotionality, can serve both as a protective factor and as a risk factor in the development of internet addiction-related issues (Brand et al., 2019). Moreover, PSMU can be considered a coping mechanism that, while occasionally utilized by individuals in offline daily life scenarios to manage situations perceived as beyond their control, tends to have more disadvantages than advantages (Marttila et al., 2021). Therefore, individuals exhibiting reduced levels of emotional intelligence may be more inclined towards employing external regulatory strategies, such as PSMU, to alleviate distress. For instance, individuals who lack the necessary skills to effectively regulate negative emotions in response to stressful situations may resort to social media platforms as a way of self-protection, to divert attention and manage uncomfortable emotions, yet ultimately experience detrimental mood alterations (Marino et al., 2020). The results, consistent with previous research findings, further substantiate this claim (Gracia Granados et al., 2020; Süral et al., 2019).

Upon further exploration of this effect, this research discovered that both PR and EA independently mediated the relationship between EI and PSMU. In regard to PR, Individuals with higher EI exhibit superior emotional regulation and are also better equipped to provide effective social and emotional support within peer groups. As a result, they tend to maintain stable relationships and build resilient interpersonal networks more effectively (Chen et al., 2023). This helps meet the individual’s needs for interpersonal relationships and emotional well-being, further promoting positive emotional experiences and behavioral responses (Mayer et al., 2016). In the absence of adequate and stable PR, individuals are more likely to develop PSMU as a coping mechanism for stress relief or as an escape from the pressures of real life, which can be interpreted as an adaptive strategy for self-protection (Hou et al., 2017; Hussain and Griffiths, 2021). This excessive reliance on online social networking may lead individuals to confine their PR and interpersonal interactions primarily within the virtual realm, resulting in a state of PSMU (Zhao et al., 2023).

However, individuals with lower levels of PR are at a higher risk for EA, which is also identified as one of the risk factors associated with PSMU, potentially exacerbating their susceptibility to developing PSMU (Beltrán-Ruiz et al., 2023). Individuals with lower levels of PR also tend to exhibit relatively deficient self-emotional regulation. When confronted with aversive stimuli or situations, they frequently resort to avoidance or escape as a coping mechanism. Virtual online social networks offer them a comparatively secure and cost-effective environment, facilitating their inclination towards immersion in online social networks as a means of evading or eluding unpleasant stimuli or situations (García Montes et al., 2024).

Furthermore, the research findings from the chain mediation analysis not only validated the applicability of the I-PACE model but also elucidated a sequential mechanism that influences PSMU (Brand et al., 2016, 2019). In fact, previous studies have also indicated that PR can serve as a predictor of EA (Wolgast et al., 2013).

Therefore, these research findings indicate that individuals with lower EI tend to experience less effective PR and impaired interpersonal communication. Furthermore, these negative experiences with peer relationships may engender an aversion to peer interactions and a propensity to avoid interpersonal connections as a means of evading unpleasant experiences. Ultimately, individuals might resort to excessive utilization of social networks as a strategy for coping with these unfavorable peer relationships and negative experiences.

This investigation examines various influential factors contributing to PSMU, thereby offering novel avenues for research and intervention strategies aimed at mitigating and preventing individuals’ engagement in PSMU. Although EI is frequently considered a stable personality trait, the intervention studies suggest that it can be enhanced through appropriate training and development programs (Rico-González, 2023; Puffer et al., 2021). Therefore, clinical and educational professionals may endeavor to facilitate individuals in improving their EI through targeted training interventions. This would enable them to effectively manage interpersonal relationships, experiential avoidance, and cope with real-life stressors while overcoming their own PSMU. Moreover, providing direct assistance to individuals for enhancing social skills and fostering positive peer relationships could serve as an efficacious approach towards reducing the occurrence of PSMU.

### 7.1 Limitations

While the results of this research provide valuable insights into understanding and elucidating PSMU, certain limitations persist. Firstly, although the sample data of this study were collected from various regions and cultural contexts within China, thereby ensuring a high degree of universality and cross-cultural applicability, it is important to acknowledge that China is home to a rich diversity of ethnic groups, making it a multi-ethnic nation with significant economic and cultural disparities across different regions. Therefore, future studies should further investigate how regional economic and cultural factors influence the relationship between EI and PSMU. Secondly, although the mediation model was grounded in theoretical foundations (Brand et al., 2019), the cross-sectional design of this study restricts its capacity to establish causal relationships between variables. To delve deeper into the potential mechanisms, future research could adopt longitudinal designs and panel studies. In addition, the reliance on self-reported assessments in this study may introduce potential bias due to social desirability effects. To mitigate this influence, future research could incorporate objective assessments of EI (Mayer et al., 2016, 2002) and clinical evaluations for PSMU.

## 8 Conclusion

This research provides empirical evidence that EI indirectly influences PSMU. This influence occurs through the mediating roles of PR and EA. The findings from the chain mediation analysis not only validated the applicability of the I-PACE model but also elucidated a sequential mechanism that influences PSMU: lower EI is associated with poorer RP, which subsequently contributes to increased EA, ultimately resulting in higher levels of PSMU. Consequently, prevention initiatives for PSMU should focus on enhancing EI, improving skills for managing PR, and reducing EA.

## Data Availability

The original contributions presented in the study are included in the article/Supplementary material, further inquiries can be directed to the corresponding authors.
