# Supplementary material for: Effect of emotional intelligence on problematic mobile social media use: mediating role of peer relationships and experiential avoidance
Source: Front Psychol. 2025 Jun 19;16:1558733. doi: 10.3389/fpsyg.2025.1558733 (PMC12222163; doi:10.3389/fpsyg.2025.1558733)
Supplement: Supplementary file 1 [file Data_Sheet_1.PDF]

# 伦理审查意见

| | | | |
|---|---|---|---|
| 审查意见号 | 成医伦审 2021 NO.07 | | |
| 项目名称 | 青少年网络成瘾行为及其影响因素研究 | | |
| 项目来源 | ☐政府 ☐基金会 ☐国际组织<br>☐企业 ☑其他: 自选课题 | | |
| 项目负责人 | 陈孜 | | |
| 送审的受理日期 | 2021.05.28 | | |
| 审查类别 | 初始审查 | 审查方式 | 快速审查 |
| 合规性声明 | 生物医学伦理委员会组成和运行遵循 GCP 和相关法律法规 | | |

**审查意见**

根据国家药监局和国家卫健委《药物临床试验质量管理规范（2020)》，WMA《赫尔辛基宣言（2013)》和 CIOMS《人体生物医学研究国际道德指南（2016)》的伦理准则，经学校生物医学伦理委员会审查，同意开展本项研究。

请遵循 GCP 原则、遵循伦理委员会同意的方案开展临床研究，保护受试者的权益与安全。

研究开始前，请申办者/研究者完成临床试验注册。研究过程中，请依规提交修正案申请，年度报告或研究进展报告，安全性报告，偏离方案报告，终止或者暂停研究报告，研究完成报告。研究过程中，发生为消除对受试者紧急危害的研究方案的偏离或者修改;增加受试者风险或者显著影响临床研究实施的改变;所有可疑且非预期严重不良反应;可能对受试者的安全或者临床研究的实施产生不利影响的新信息，请及时报告生物医学伦理委员会。

| | |
|---|---|
| 年度/定期审查频率 | 24 个月 |
| 起止日期 | 2019 年 12 月 26 日——2021 年 12 月 26 日 |
| 同意研究的有效期 | 24 个月 |
| 起止日期 | 2019 年 12 月 26 日——2021 年 12 月 26 日 |
| 伦理委员会签发者 | [illegible]（签字） |
| 签发日期 | 6/2/2021 |

成都医学院生物医学伦理委员会

# Ethical review opinions

| | | | |
|---|---|---|---|
| **Review opinion number** | Chengdu Medical College EC 2021 NO.07 | | |
| **project name** | Internet addiction behavior and its influencing factors among adolescents | | |
| **Project source** | □ Government □ Foundation □ International Organization<br>□ Enterprise ☑ Other: self-selected topic | | |
| **project leader** | Zi Chen | | |
| **The acceptance date of the submission for trial** | 2021.05.28 | | |
| **Review category** | Initial review | **Review method** | Quick review |
| **Compliance Statement** | The biomedical ethics committee was composed and operated in accordance with the GCP and relevant laws and regulations | | |

**Review opinions**

By the ethical guidelines of the State Drug Administration (SDA) and the National Health Commission (NHC) Code of Practice for the Quality Management of Drug Clinical Trials (2020), the WMA Helsinki Declaration (2013), and the CIOMS International Ethical Guidelines for Biomedical Research on Human Subjects (2016), and after reviewing by the University's Biomedical Ethics Committee, consent was granted for the conduct of this study.

Please follow the GCP principles and the protocol agreed upon by the Ethics Committee to conduct the clinical research and protect the rights and safety of the subjects.

Before the commencement of the study, sponsors/investigators are requested to complete the clinical trial registration. During the survey, submit an amendment application, annual report or study progress report, safety report, protocol deviation report, termination or suspension report, and study completion report as required. During the study, please report to the BioMedical Ethics Committee any deviation or modification of the study protocol to eliminate an imminent hazard to subjects; any change that increases the risk to subjects or significantly affects the conduct of the clinical study; all suspected and unanticipated serious adverse reactions; and any new information that may adversely affect the safety of the subjects or the conduct of the clinical research.

| | |
|---|---|
| **Annual / periodic review frequency** | 24 Months |
| **Start and end date** | 26 Dec 2019 —— 26 Dec 2021 |
| **Consent to the validity period of the study** | 24 Months |
| **Start and end date** | 26 Dec 2019 —— 26 Dec 2021 |
| **Issued by the ethics committee** | Ying Xu (sign) |
| **Date of issue** | 6/2/2021 |
